# Intrinsic Asymmetry in Weak Acid Transmembrane Transporters

**DOI:** 10.3390/biom16010091

**Published:** 2026-01-06

**Authors:** Emmi Jaeger, Sebastian Buss, Eric Beitz

**Affiliations:** Pharmaceutical Institute, Kiel University, Gutenbergstr. 76, 24118 Kiel, Germany; ejaeger@pharmazie.uni-kiel.de (E.J.); sbuss@pharmazie.uni-kiel.de (S.B.)

**Keywords:** transmembrane transport, asymmetry, channel, facilitator, proton, monocarboxylate, lactate, Warburg effect, aquaporin, basigin

## Abstract

Transmembrane facilitation of substrates by channels and secondary active transporters results in a defined steady-state concentration ratio across the membrane. Evidence is accumulating that asymmetry in the structural build of the transporters, or interaction with asymmetric partner proteins, can shift the position of the transmembrane equilibrium by biased transport directionality. For instance, the bacterial lactose transporter, LacY, and two amino acid transporters, i.e., the human excitatory amino acid carrier, EAAC1, and the yeast lysine permease, Lyp1, were reported to exhibit distinct transport kinetics in the inward and outward direction by protein-intrinsic properties. A recent example is transport modulation of human monocarboxylate transporters, MCT, by shedding of the extracellular domain of an ancillary protein, basigin. Loss of the domain selectively increases export of lactate from lung cancer cells by a factor of four, contributing to the Warburg effect and malignancy. Further, intrinsic properties of monocarboxylate transporters involving asymmetric affinities of substrate binding, or biased open probabilities were shown to generate preference for one transport direction. Here, we discuss molecular mechanisms and physiological contexts of asymmetric secondary active transmembrane transport. Focus is laid on experimentally established cases, and examples are given in which putative bias in transport directionality may have been overlooked.

## 1. Introduction

**Figure 1:**
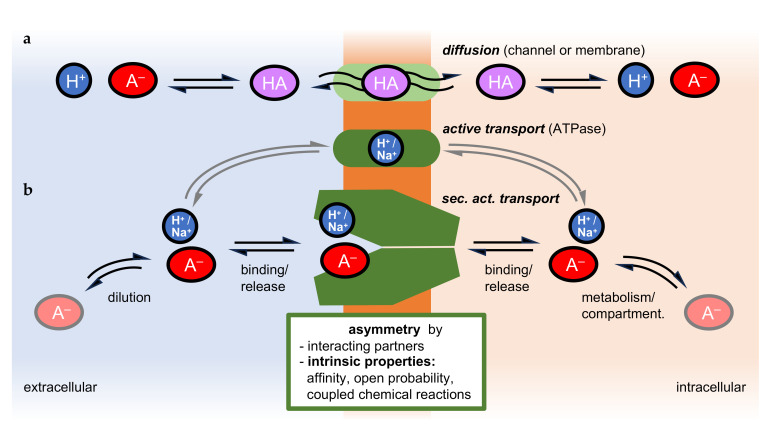
Transmembrane translocation of weak acids by (**a**) diffusion and (**b**) secondary active transport. Transport is mainly driven by transmembrane gradients. Membrane proteins can generate asymmetry by directional transport bias.

Transport of molecular substrates across cellular membranes is the foundation of compartmentalized metabolism [1]. The translocation of nutrients should preferably occur in the import direction, and that of waste products outward [2]. Transport is driven by transmembrane substrate gradients that build up due to biochemical reactions. The passive diffusional permeability of the lipid membrane alone for polar or charged metabolites, however, is particularly low and would not suffice to enable fast enough passage to maintain metabolic pathways of complex organisms (Figure 1a). Therefore, channel and transport proteins facilitate transmembrane translocation by providing more suitable and efficient passageways (Figure 1a,b). Next to transmembrane substrate gradients, additional electrochemical and pH gradients can drive translocation by using Na^+^ or H^+^ as cosubstrates [3,4,5]. Large ion or proton gradients can propel substrates even against their concentration gradient.

Transmembrane translocation via channels and transporters reaches an equilibrium when the substrate and cosubstrate gradients are balanced. The steady-state transmembrane concentration ratio is termed the equilibrium position. Under these conditions, the free energy in the system is minimized. Disturbances of the steady-state concentration ratio, e.g., accumulation of substrates to higher concentrations, can only be achieved by an input of energy into the system. For this, primary active transporters, ATPases, derive chemical energy from hydrolysis of ATP, which is used to establish steep transmembrane electrochemical (e.g., a cell’s resting potential) [6,7,8] or proton gradients (e.g., acidic organelles) [9,10] (Figure 1b).

Recent evidence indicates that channels and secondary active transporters can also shift the transmembrane equilibrium position of the transported substrate by intrinsic mechanisms [11,12,13] or with the help of partner molecules [14,15] (Figure 1b). This phenomenon is brought about by asymmetric protein properties. Even though the degree by which the steady-state concentration ratio can be shifted by channels and secondary active transporters is smaller than what is achievable by ATPases, physiological relevance has been shown [15,16,17]. In this review, we discuss experimentally established cases of preferred transport directionality of channels and transporters due to intrinsic properties, and underlying principles with a focus on the transmembrane translocation of weak acids.

## 2. Transmembrane Equilibrium of Weak Acids

### 2.1. Weak Acid Dissociation Equilibrium

Weak acids are characterized by a dissociation constant, p*K*_a_, close to the physiological pH, e.g., acetic acid (p*K*_a_ 4.8) or lactic acid (p*K*_a_ 3.8) [18]. The proportions of the protonated, neutral acid molecule, [HA], and the deprotonated, negatively charged acid anion, [A^−^], at a given pH can be calculated using the Henderson–Hasselbalch equation [19], pH = p*K*_a_ − log([HA]/[A^−^]). For instance, acetic acid dissociates, [HAc] D [H^+^] + [Ac^−^], in an aqueous solution of pH 7.3 by 99.68% into the acid anion and a proton, whereas 0.32% remain as the neutral acid species. For lactic acid at pH 7.3, due to a ten-times-higher acid strength, this ratio is shifted by one order of magnitude towards dissociation, resulting in 99.97% anions and 0.03% neutral acid. The p*K*_a_ depends on the dielectric properties of the solvent. Free bulk water due to a high relative permittivity (or dielectric constant) ε typically around 80 strongly promotes weak acid dissociation by hydration of the released ions, whereas less hydrophilic media such as the interior of the lipid membrane lower the acid strength and degree of dissociation (ε as low as 10) [20]. Water in confined protein environments or surface-bound water loses its continuum properties, resulting in highly variable ε [21,22]. Further, due to the different hydration properties of the dissociated and undissociated acid, both agents tend to distribute unequally if a non-aqueous interface, e.g., air or potentially a protein surface, is present, leading to a relative accumulation of the electroneutral acid at the surface, whilst the anion is primarily present in the bulk water [23]. The process of protonation/deprotonation is dynamic even when the equilibrium state has been reached, meaning that a given molecule undergoes rapid changes in its protonation state while the general ratio of neutral acid and anion remains constant. Disturbance of the equilibrium by removal of molecules of one species, for instance, by diffusion of neutral acid across a membrane [24] (Figure 1), will be compensated immediately by the protonation of acid anions to re-establish the dissociation proportions. Another consequence of pH-dependent weak acid dissociation is that a transmembrane pH gradient will lead to accumulation of the weak acid in the less acidic compartment [25]. For example, a gradient of 0.3 pH units can produce a 2-fold enrichment, and a gradient of 1 pH unit a 10-fold accumulation. This ion trap principle drives compartmentalized metabolism [26] and is used in drug development to modulate pharmacokinetic properties [27].

### 2.2. Transmembrane Transport of Weak Acids Affects Compartment pH

Transmembrane translocation of neutral, protonated weak acid molecules will shift the pH of the delivering compartment towards alkaline (loss of protons) [28,29] and the receiving compartment towards acidic (gain of protons) [30,31]. Changes in the proton concentration can be measured using pH electrodes (in unbuffered or weakly buffered extracellular solution or intracellularly using microelectrodes [32]) or pH-sensitive fluorescent sensor molecules, such as pyranine [33] or carboxyfluorescein [30,31]. In a physiological setting, disturbances in the proton concentration are typically corrected by the activity of primary active H^+^-ATPases that pump protons across the membrane [9] (Figure 1). As translocation of weak acids, HA or H^+^/A^−^ [24], is electroneutral, the electrochemical membrane potential is irrelevant (for Na^+^-coupled acid anion transport, see Section 3.4). However, additional driving forces for weak acid transport are, for instance, dilution in the extracellular compartment, e.g., by the blood circulatory system, and metabolic conversion or compartmentalization in cellular organelles (Figure 1).

### 2.3. Membrane Proteins Facilitate Translocation of Weak Acids

Different types of membrane proteins facilitate transmembrane translocation of weak acid substrates (Figure 1). Among these are strict channels for neutral acids (certain aquaporin isoforms [29,34,35,36]), or channel-like proteins from the microbial formate-nitrite transporter family [37,38,39,40,41] that conduct acid anions at neutral pH [42] and can switch to acid anion/H^+^ cotransport at acidic pH [39,43]; both protein types are members of the major intrinsic protein superfamily, MIP [44]. Secondary active transporters of the major facilitator superfamily, MFS, use Na^+^ or H^+^ gradient-driven mechanisms of weak acid anion cotransport [45].

Generally, such membrane proteins are thought to increase the velocity of transport in both directions. This is accomplished by providing membrane-spanning proteinaceous environments that are more suitable for accommodating weak acid molecules in their anionic or neutral form than the lipophilic interior of the lipid bilayer. The role of the facilitator protein can be seen as that of a catalyzer in a chemical reaction, which would accelerate the establishment of an equilibrium but would not affect the equilibrium position [46]. This is achieved by lowering the activation energy equally for both the forward and reverse reaction. Does this view apply to transmembrane facilitators? In other words, could such proteins be able to bring about asymmetry by preferring transport in one direction over the other irrespective of secondary driving forces? Some examples provide experimental evidence for biased transport directionality of secondary active transporters. Explanations for the observed asymmetry involve protein-intrinsic properties or contributions by interaction partners. The scarcity of data is probably due to the requirement of a demanding assay setup that ensures determination of the true inward and outward transport kinetics, or the undisturbed equilibrium position by excluding secondary effects such as electrochemical gradients or substrate loss by metabolism or compartmentalization.

### 2.4. Transmembrane Equilibrium State

At equilibrium, the inward, *k*_in_, and outward transport velocities, *k*_out_, are balanced (*k*_in_ = *k*_out_), yielding the equilibrium constant *K*_eq_ [14]. For proton-coupled anion translocation, this means that a stable, albeit dynamic ratio of the external and internal proton and anion concentrations has been achieved, *K*_eq_ = [H^+^]_e_ [A^−^]_e_/[H^+^]_i_ [A^−^]_i_, and the free energy of the system, i.e., the Gibbs energy *ΔG*, equals 0. At equal pH at both sides of the membrane, the acid anion concentration would be even, and a transmembrane pH gradient would shift the anion concentrations accordingly.

Transporters that translocate their substrate in an asymmetric fashion would establish a transmembrane concentration ratio that differs from the purely diffusion-controlled equilibrium position, and, consequently, *ΔG* would not reach 0. Primary active transporters gain the energy that is required for asymmetric transport from hydrolysis of ATP anhydride bonds [6,7,8,9,10]. This way, steep electrochemical gradients in the order of −100 mV can be built up across the membrane. Channels and secondary active transporters do not have comparable means to gain high levels of energy from coupled chemical reactions. Protein-intrinsic properties that may contribute to biased transport directionality would, therefore, exhibit effects at a lower level than primary active transporters can achieve. Accordingly, asymmetry in secondary active transport may have been widely overlooked. However, a rise in awareness may be of value because physiological relevance has been shown even for small biases in directional transmembrane transport.

For instance, a fourfold shift in the position of the lactate transmembrane equilibrium toward export renders lung carcinoma cells more malignant [15,16]. Here, monocarboxylate transporter 4, MCT4, exhibits different import/export kinetics in the presence and absence of the extracellular domain of an ancillary protein, basigin, upon cleavage by a protease [15,16,17]. The difference in free energy, *ΔΔG*, that accounts for a fourfold shift in the transmembrane equilibrium between MCT4 with an associated full-length basigin, *K_eq__*_full_, and a truncated variant, *K_eq__*_trunc_, amounts to *ΔΔG* = *R T* ln (*K*_eq_*___*_full_/*K*_eq_*___*_trunc_) = 3.4 kJ mol^−1^ [47], with *R* being the gas constant, 8.314 J mol^−1^ K^−1^, and *T* being the absolute temperature (292 K in the assay). This is one-tenth of the energy that is released by ATP hydrolysis [48].

### 2.5. Asymmetry-Generating Intrinsic Properties of Channels and Facilitators

Other than chemical catalyzers, transmembrane-spanning proteins expose different faces to substrate molecules approaching from the extra- or intracellular side. While channels of the MIP superfamily are rigid tube-like proteins with two open ends [44] (gating ignored), transporters of the MFS superfamily [45] present only one open substrate entry site at a time, and undergo a major conformational conversion upon binding of the substrate, termed alternating access mechanism [3,4,5]. Further, the substrate binding affinity of channel proteins is typically very low, accommodating swift passage [28], whereas transporters can achieve (sub-)micromolar substrate affinities via electrostatic, hydrogen bond, or lipophilic interactions [3,4,5]. Therefore, there are likely fewer options for channels to generate protein-intrinsic asymmetry than for transporters.

**Figure 2:**
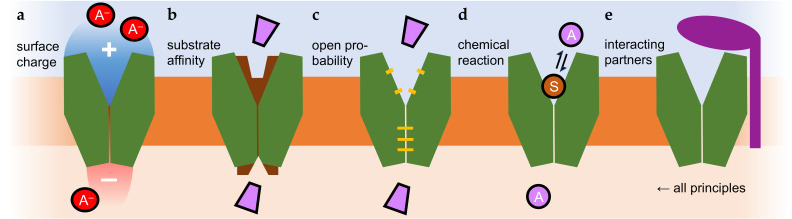
Asymmetry-generating properties of transmembrane translocation proteins. Surface charge (**a**) and substrate affinity (**b**) can increase local substrate concentrations. The open probability may be biased when one state is energetically more favorable (**c**), and coupling transport to chemical reaction energy can drive asymmetric translocation (**d**). Asymmetric interaction or fusion with partner proteins can promote all named modes of biased transport (**e**).

Intrinsic asymmetry in channel and transporter activity, however, appears possible and has been shown experimentally (Figure 2). Respective protein-intrinsic properties may be brought about by differences in the affinity and accessibility of the substrate binding site. Here, differences in the extra- and intracellular electrostatic surface charges affecting conformational changes (Figure 2a), the substrate affinity (Figure 2b), or open probabilities (Figure 2c) may contribute to asymmetry. For weak acid (and weak base) transporters, protonation/deprotonation reactions involving the substrate itself have been proposed to take place in the transporter vestibules [39,43,49] that may couple chemical potential energy to transmembrane translocation (Figure 2d). Another possibility is interactions with protein partners [14,15,16] or small molecules [50] that occur only on one side of the membrane, and can confer any of the named principles (Figure 2e).

## 3. Asymmetry in the Translocation of Charged Cargo

For transmembrane translocation of permanently charged substrates, the membrane resting potential is the major driving force and primarily determines transport directionality [51]. Only very few examples of protein-intrinsic properties have been found to counteract this force to some degree. The typical electric potential across the cell membrane is negative in the cell interior, usually in the range of −60 to −80 mV, compared to the extracellular space. This means that the electrical gradient drives positive charges into the cells, and negative charges out. It can only be overwhelmed by very steep chemical substrate gradients, or by coupling the translocation process with the cotransport of ions that generate a suitable net charge of the combined substrate/cosubstrate. Additionally, the lipid membrane itself, due to the presence of charged polar head groups (e.g., negative phosphate or positive ethanolamine), has electrostatic and polarity properties that contribute the transmembrane translocation of charge [52,53].

### 3.1. Inward-Rectifying Potassium Channels, Kir (K^+^)

The classical example for asymmetric channel activity is preferred K^+^ influx via inward-rectifying potassium channels, Kir [54]. Inward rectification remains even when the electrical transmembrane gradient is reversed, i.e., additional asymmetric factors must be in place. It was found that the intracellular K^+^ channel entry site tends to bind and be blocked by other positively charged inorganic, Mg^2+^, or organic polyamine cations such as spermine [50]. The intracellular channel face, with the help of endogenous cations, thus, acts like a one-way pressure valve by allowing K^+^ ions to flow in but preventing them from easily permeating outward (Figure 2e). Lower levels of rectification in ion channels can also be derived from intrinsic charge distribution properties (Figure 2a); see, for instance, [55]. A large body of literature is available on ion channel structure–function relationships that cannot be addressed within the scope of this paper.

### 3.2. The Prototypical Secondary-Active Lactose Permease, LacY (Neutral Galactoside + H^+^)

**Figure 3:**
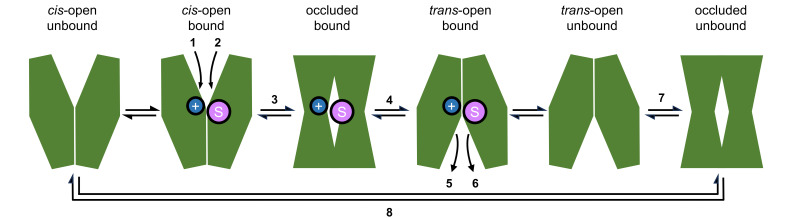
Principle mechanism of secondary-active transport. Eight events involving binding and release of the cosubstrate (blue) and the substrate (magenta), and subsequent conformational changes lead to different states. See text for details.

Protein structural data on several conformational states and functional analyses of secondary-active transporters, e.g., of the *Escherichia coli* lactose permease, LacY [11,56], provided detailed insight into the basic alternating-access transport mechanism (Figure 3). Initially, a proton (step 1) and the neutral sugar (step 2) bind to the open *cis*-cavity of the transporter in a coordinated fashion. Binding initiates a large conformational change, which occurs through an occluded, substrate-bound state (step 3) to the *trans*-open conformation (step 4). Subsequently, the proton (step 5) and the sugar (step 6) are released. The transporter can transition back via an occluded, unbound-state (step 7) to the initial *cis*-open state (step 8). Each step in the process bears potential for asymmetry. The growing body of structural information on secondary-active transporters yielded three models for the conformational transitions during transport, termed rocker switch, rocking-bundle, and elevator mechanism, which are reviewed elsewhere [5,57,58,59].

The physiological scenario for LacY in bacteria is proton-driven assimilation of sugar from the environment as a nutrient [11,56]. However, as all steps in the transport mechanism are reversable (Figure 3), LacY generally acts bidirectionally, and high lactose concentrations can force uphill proton movement. Nevertheless, import via LacY is prioritized not only by an inward proton gradient in the cellular context but also by a 50–100-times-higher apparent lactose affinity for import than for export upon protonation of Glu325 (see Figure 2b) [11]. The pK_a_ of the Glu325 sidechain is dramatically shifted by six orders of magnitude to 10.5 due to the low permittivity of the protein environment. A LacY mutant in which Glu325 was replaced by neutral alanine was still capable of translocating galactoside, yet independently of transmembrane pH gradients [11].

Moreover, experiments on substrate binding to LacY with a single-sidedly sealed cavity by an artificial disulfide bridge indicated asymmetrical transitions between inward and outward conformations. Despite a closed periplasmic cavity, the cytosolic substrate binding site still switched between occluded and open states in a two-step process. This may lead to uneven open probabilities at the two sides of the transporter (Figure 2c) [60].

### 3.3. Excitatory Amino Acid Carrier, EAAC1 (Glu^−^ + 3 Na^+^)

Another experimentally validated case of asymmetric substrate translocation partially counteracting the electrochemical gradient is that of the neuronal excitatory amino acid carrier 1, EAAC1 [61]. Here, glutamate transport is coupled to the cotransport of three sodium ions [12]. The net charge of the acidic amino acid with a negatively charged carboxyl at the sidechain plus 3 Na^+^ is +2. Transport in the inward direction is driven by the steep electrochemical sodium gradient, which largely overcompensates the repulsion of the negative charge of glutamate by the negative resting membrane potential. It came as a surprise that under these conditions EAAC1 is also capable of transporting glutamate out of the cells when the glutamate gradient is reversed. A comprehensive electrophysiology study found different kinetic parameters for the inward and outward transport of glutamate. While glutamate import was strongly voltage-dependent but relatively slow, export was less voltage-dependent and considerably faster. A first-in-first-out model with respect to one of the three Na^+^ ions was proposed. Here, binding of Na^+^ before the glutamate substrate and release of the Na^+^ in the receiving compartment before glutamate would explain the observed asymmetric transport properties [12].

### 3.4. Lysine Permease, Lyp1 (Lys^+^ + H^+^)

Inward transport of positively charged amino acids such as lysine is promoted by the negative membrane potential. The yeast lysine permease, Lyp1 [62], is further coupled to proton cotransport, i.e., an inward pH gradient additionally drives lysine uptake into the cells [63]. Lyp1 activity results in a quasi-irreversible intracellular accumulation of up to 70 mM lysine, which is maintained in the absence of extracellular lysine and even upon dissipation of the inward-directed proton motive force [13]. Directional transport kinetic properties were determined by reconstituting purified Lyp1 protein in proteoliposomes to avoid secondary effects by cellular processes and compartmentalization of lysine in the acidic yeast vacuole. This brought about large differences of 3–4 orders of magnitude in substrate affinity for the extra- and intracellular transporter entry sites. Binding to the extracellular side and import yielded a micromolar apparent *K*_m_, whereas *K*_m_ in the outward direction was estimated to be millimolar (Figure 2b). Analysis of structure–function relationships in Lyp1 identified sections in the extracellular loops of the alternating access transporter that appeared to be responsible for the asymmetry in substrate binding [64].

### 3.5. Sodium-Coupled Monocarboxylate Transporters, SMCT (Monocarboxylate^−^ + 2 or More Na^+^)

Transmembrane transport of monocarboxylates is either coupled to the cotransport of protons (Section 4) or sodium cations. Sodium-coupled monocarboxylate transporters, SMCTs, are mainly expressed in the kidney and intestine, where they make use of the inward Na^+^ gradient to transport a variety of monocarboxylates and short-chain fatty acids into the epithelial cell layer. Transport via the high-affinity isoform SMCT1 (sub-millimolar *K*_m_) is clearly electrogenic, indicating cotransport of at least two Na^+^ cations per monocarboxylate anion, leading to a net positive charge being transported into the cell [65,66]. The case is less clear for SMCT2, which exhibits 35–80 times lower substrate affinity. Here, monocarboxylate transport is accompanied by low currents that are still casting uncertainty regarding its electrogenicity, and hint at a rather even monocarboxylate/Na^+^ transport stoichiometry [67,68]. Lactate reabsorption in the kidney proximal tubules by SMCT1 and SMCT2 is thought to be responsible for the almost complete lack of renal lactate excretion, illustrating the significance of the inward-directed electrochemical Na^+^ gradient as a driving force [67]. Another consequence of coupling monocarboxylate transport with Na^+^ cotransport is that the acid-anion-associated proton is left behind in the primary urine. Separation of the acid anion and the proton may be a necessity for operating the sophisticated pH regulation mechanisms of the kidney that maintain the acid–base balance of the body.

## 4. Asymmetry in Proton-Coupled, Net-Neutral Weak Acid Transporters

Contrary to transmembrane translocation of charged cargo, proton-coupled weak acid transport is electroneutral rendering the membrane potential irrelevant. Further, besides having separate binding sites for substrate and cosubstrate, evidence from evolutionary and functionally unrelated weak acid anion/H^+^ facilitating protein families indicates a common theme: at some point in the translocation process, the protonated neutral acid is formed as an intermediate or as the actual transported species. The typically lower relative permittivity within the protein environment or effects of confined or surface-bound water promotes weak acid anion protonation. Involving the substrate itself as a proton acceptor has two consequences. First, selectivity is enhanced because inter-convertibility between charged and uncharged states is a unique physicochemical property of weak acids (and bases) that is absent in other substrates. Second, the chemical reaction energy of the proton transfer may be used to enhance transport or generate asymmetry.

### 4.1. Aquaporins with Permeability for Weak Acids

**Figure 4:**
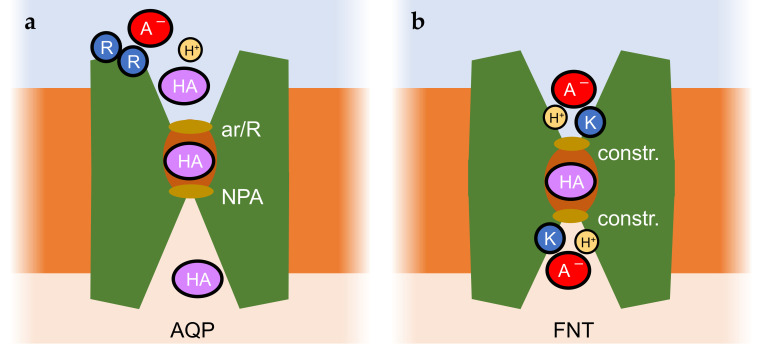
Proton-coupled weak acid transport via major intrinsic proteins, MIPs. (**a**) Aquaporins with solute permeability facilitate weak acid permeability. Shown is a protomer of the AQP homotetramer. The aromatic/Arg selectivity filter and the central Asn-Pro-Ala region (NPA) are labeled. Strong extracellular surface charges have been shown to enhance weak acid permeability. (**b**) Microbial formate-nitrite transporters, FNTs, exhibit a dielectric slide mechanism. Shown is a protomer of the FNT homopentamer with its two lipophilic constriction sites. Weak acid anions enter increasingly lipophilic vestibules by attraction by a Lys residue. The dielectric environment decreases the acid strength and promotes protonation. The neutral acid can then pass the lipophilic constriction sites and core of the transport path.

Aquaporins are water and solute channel proteins [69,70] that strictly exclude charges due to the presence of two filter regions in the permeation path, namely, the aromatic/Arg selectivity filter and the central Asn-Pro-Ala region, NPA [71,72,73,74] (Figure 4a). Hence, weak acids, e.g., lactic acid [29,34] or arsenous acid [75], and bases, e.g., ammonia [73,76,77,78], can only pass in their neutral forms.

Permeability for lactic acid was found to be enhanced by aquaporins with strongly positive surface charges due to the presence of Lys/Arg clusters [29,34]. Such charge accumulations are typical for aquaporins from lactic acid bacteria [34] but are also present, for instance, in mammalian aquaporin 9 [25,29,79,80]. Close to the positive aquaporin protein surface, the p*K*_a_ of weak acid substrates appeared shifted by up to 1 log unit, leading to higher local concentrations of the neutral substrate species, which may explain the observed increase in permeability (Figure 2a) [29]. By facilitating transmembrane diffusion of only neutral weak acid molecules, aquaporins act as elements of the ion trap mechanism [23].

A detoxifying role for arsenite was shown for certain aquaporins [75]. Arsenite is the anion of the very weak arsenous acid As(OH)_3_ (p*K*_a_ 9.2; As^III^) which mainly remains undissociated at neutral pH, rendering it a substrate of aquaporins with a wide enough pore diameter. Arsenite is derived from arsenate, i.e., the anion of arsenic acid, H_3_AsO_4_ (p*K*_a_ 2.2; As^V^), which is strongly dissociated at physiological pH. As a chemical analog of phosphate, it can enter cells through phosphate transporters. The required chemical reduction from As^V^ to As^III^ for extrusion is catalyzed by arsenate reductase enzymes [81,82]. Genetic fusions of aquaporin channels with catalytic arsenate reductase domains were identified in certain actinobacteria and found to efficiently confer arsenate resistance [83]. Tuberculosis-causing mycobacteria express a similar fusion consisting of a transport unit termed arsenic compound resistance membrane protein, ACR3 [84], and an arsenate reductase. Such fusions of channels or transporters with substrate-generating enzymes are quite unique. Generation of the permeating substrate in place for transmembrane translocation increases the local concentration in an asymmetric fashion promoting export (Figure 2d,e).

Both mechanisms involving lactic and arsenous acid facilitation by aquaporins were shown on the protein functional and physiological level. However, the true inward and outward kinetics or the transmembrane substrate equilibrium position under steady-state conditions have not yet been addressed experimentally.

### 4.2. Microbial Formate-Nitrate Transporters, FNT

Members of the formate-nitrite transporter family, FNTs [37,38,85], can be seen as an evolutionary intermediate between rigid channels and conformation-changing secondary active transporters. Substrates are small weak monoacids of inorganic (nitrous acid [85], hydrogen sulfide [86]) and organic nature (monocarboxylates [42,87]). The channel-like build of the exclusively microbial FNTs is symmetrical with respect to the plane of the membrane (Figure 4b). Both entry sides are permanently accessible. However, FNTs mostly act by a proton-driven weak acid anion/H^+^ cotransport mechanism [28,39,43]. In the absence of a transmembrane proton gradient, acid anions can leak through the FNTs [40], yet upon acidification, more efficient anion/H^+^ cotransport takes over [28,43,88]. A “dielectric slide” mechanism is made responsible for this feature [39]. The vestibules funnel the substrate to the center of the translocation path, which is shielded on either side by lipophilic constrictions. A conserved Lys residue is located deep inside each vestibule. Its positive charge attracts weak acid anions into the increasingly lipophilic protein environment. Concomitantly, the acid strength of the substrate decreases due to the increasingly lipophilic protein environment, promoting protonation. The formed neutral acid species can pass two lipophilic constriction sites that shield the core of the transport path.

Depending on the dielectric properties defined by the width, depth, and lipophilicity of the two vestibules, the processes of substrate attraction and protonation may differ at both entry sites and may thereby induce asymmetry in transport (Figure 2a,d). In fact, one report describes a point mutation that even generates unidirectional transport (export direction) via an FNT from *Escherichia coli* [89]. A mechanism involving a central and strongly conserved histidine as a checkpoint was recently proposed based on a newly resolved structure of a mutant FNT and molecular dynamics simulations [90]. Yet again, quantitative comparisons of the inward and outward transport kinetics of the FNTs or investigations of the steady-state transmembrane substrate concentrations are missing. Still, there is physiological evidence for specific roles of the various FNT with respect to preferred uptake or release of their weak acid substrates. In bacteria, formate is released as a metabolic product of mixed acid fermentation; at low external pH, formate re-uptake occurs to form CO_2_ and H_2_ catalyzed by formate lyase; the genes encoding the FNT, here termed FocA, and the lyase are organized as an operon [42]. Bacteria typically express a second FNT with a preference for nitrite, NirC, for which a pH dependence of the transport directionality is not reported. In fact, electrophysiological evidence indicates that NirC activity is only mildly affected by pH. Respiratory nitrite ammonification is a mechanism of enteric bacteria, which involves import of nitrite via NirC and chemical reduction by a coinduced reductase [91]. In addition, nitrite uptake and subsequent reduction appears to be a mechanism of *Salmonella* species to block activation of infected macrophages, thus gaining virulence through enhancing resistance against the innate immune system [92]. Further, transport of hydrogen sulfide, HS^−^/H^+^ (p*K*_a_ 6.8), by FNT3 is thought to provide access to environmental sulfur (uptake) but may also keep intracellular hydrogen sulfide concentrations at non-toxic levels (release) [86]. FNTs from eukaryotic protozoa [28,93,94] contain widened selectivity filters that accommodate larger substrates up to lactic acid [95]. The genomes of malaria-causing *Plasmodium* species, for instance, encode a single FNT [28], which is vital for the parasites. Release of the metabolic product lactate/H^+^ maintains the energy generation of the parasites and prevents acidification of the cytosol [96]. It was validated as a novel antimalarial drug target [97].

### 4.3. Mammalian Monocarboxylate Transporters, MCTs

#### 4.3.1. MCT-Intrinsic Properties Generating Asymmetry of Transport

Mainly, four MCT isoforms, MCT1–4 [98,99,100,101,102], are responsible for proton-coupled cellular import and export of monocarboxylates including pyruvate, ketone bodies, and lactate, e.g., in tumorous tissue [103], the neuron-astrocyte shuttle [104], or between white and red muscle fibers [105]. MCT1 and MCT2 exhibit higher affinities for lactate (low or even sub-millimolar *K*_m_ measured in the import direction [14,106]) than MCT3 and, particularly, MCT4 for which a *K*_m_ around 30 mM was determined [107]. The substrate affinities of the MCTs can be associated with roles in their physiological environments. MCT1 is present in cell types with preferred lactate import as well as in exporting cells, while MCT2 is found in cell membranes that mainly import, and MCT3/MCT4 that export, lactate [102].

**Figure 5:**
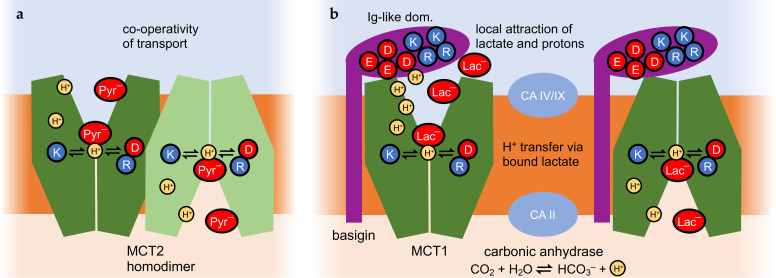
MCT interaction partners. The general MCT transport mechanism involves a proton transfer across the bound substrate. Different dielectric properties of the extra- and intracellular vestibules may affect this chemical reaction. (**a**) MCT2 homodimers exhibit cooperativity of pyruvate transport. (**b**) Asymmetric lactate transport via human MCT1. Import is promoted by the extracellular domain of basigin. Carbonic anhydrases, CAs, at the extracellular (isoforms IV and IX) and intracellular side of the membrane (isoform II) may generate bias by handling protons differently.

As a common feature of the MCT transport mechanism, a proton-relay function of the bound weak acid substrate was proposed (Figure 5) [49]. Transfer of a proton from a conserved Lys (position 38 in MCT1) across the monocarboxylate substrate to a stabilizing Asp/Arg salt bridge initiates the switch between the outside- and inside-open conformations. The protonation and deprotonation events possibly depend on the lower permittivity due to lipophilicity or confined water structures in the extracellular and intracellular vestibules in a similar fashion to the FNTs (Figure 4b) [39,43], and may generate asymmetry in transport.

The first evidence shows that MCT-intrinsic properties alone can generate direction-biased transport, i.e., when all other influences, such as pH gradients, metabolism, and interacting proteins, are eliminated [49]. *Saccharomyces cerevisiae* yeast was used as a system for expressing and assaying lactate transporters [108]. Yeast as an alcoholic fermentation organism does not metabolize lactate under normal growth conditions [14,25,49]. Therefore, a yeast knockout strain lacking the endogenous monocarboxylate transporters Jen1 and Ady2 allows the monitoring of undisturbed transmembrane MCT lactate transport until a transmembrane equilibrium is reached (typically within 30 min) [14]. The equilibrium position is independent of the number of transporters in the plasma membrane, enabling comparisons of different expression constructs. Replacement of the proton-accepting Lys38 in MCT1 by a neutral amino acid residue maintained transport activity; however, the pH dependency of uptake was largely shifted toward acidic extracellular pH conditions [49]. In addition, the capacity of transport or equilibrium position was altered with the mutation [49], indicating that inward and outward transport kinetics are defined by residues of the monocarboxylate binding site.

#### 4.3.2. MCT2 Homodimerization and Cooperativity of Transport

Cryo-electron microscopy structure elucidation of the MCT2 protein revealed homodimerization (Figure 5a) [109]. The transport kinetics of the MCT2 homodimers were analyzed for pyruvate export using an FRET-based substrate biosensor. Assaying transport at increasing pyruvate concentrations showed a non-linear behavior with a Hill coefficient of 1.6, indicating cooperativity of transport [109]. Possibly, the conformational change of one MCT2 protomer facilitates that of the partner protein via contacts in the transmembrane domains. However, it is not yet known if cooperativity within the monocarboxylate MCT2 homodimer brings about asymmetric transport properties.

#### 4.3.3. Asymmetric Interactions of MCT with Basigin and Possibly Carbonic Anhydrases

Two ancillary proteins have been identified to interact with MCTs, namely, basigin and embigin [110,111]. These are single-transmembrane domain proteins with the major portion being located extracellularly and consisting of glycosylated immunoglobulin-like domains, Ig, whereas the intracellular C-terminus is rather short (Figure 5b) [112,113]. Basigin association occurs mainly with MCT1, MCT3, or MCT4, and embigin with MCT2 [110]. A crucial role of basigin or embigin is in the intracellular trafficking of MCTs to their site of action, the plasma membrane [114,115].

Besides its classical function in MCT trafficking, basigin was recently shown to modulate lactate transport also directly [14,15,16,17]. The first evidence came from the identification of a transmembrane protease, TMPRSS11B, that is capable of cleaving basigin close to the extracellular face of the plasma membrane [15]. In lung carcinoma cells, shedding of the basigin Ig-like domains enhances lactate export via MCT4 by about fourfold [15]. Release of lactate from tumor cells is an indicator of aerobic glycolysis, termed the Warburg effect [116]. Here, despite a sufficient supply of oxygen, the glycolytic product pyruvate is not used to drive mitochondrial oxidative phosphorylation. Instead, it is chemically reduced via lactate dehydrogenases to form lactate. Secreted lactate can promote the growth of neighboring cells in the tumor that undergo oxidative phosphorylation. The increased release of lactate from Warburg cells after basigin shedding was found to increase tumor cell malignancy [15,16].

The Ig-like domains of basigin were shown to induce asymmetry in the MCT1 transport kinetics [14]. To this end, fusion proteins were generated, consisting of basigin variants and MCT1, and lactate transport kinetics were determined in yeast. A fourfold shift toward export in the transmembrane equilibrium position was obtained when the membrane-vicinal Ig-like domain of basigin was absent or misfolded [14]. Calculation of the charge distribution in the Ig-like domain revealed a strongly positive surface patch next to a strongly negative one. This hints at a putative role of the Ig-like domain in the recruitment and local accumulation of lactate anions and protons close to the extracellular MCT entry site (Figure 5b). The locally elevated lactate/H^+^ concentrations may enhance import [14], whereas shedding of the Ig-like domain eliminates this effect [15].

Eventually, the activity of carbonic anhydrase enzymes was linked to monocarboxylate transport via MCTs [117]. The catalyzed reaction is conversion of CO_2_ and water into bicarbonate and a proton (Figure 5b), and vice versa. The release or use of protons in the reaction may couple to nearby proton-driven transmembrane transport, particularly in the case of membrane-bound isoforms of the carbonic anhydrases, i.e., intracellular isoform II [118,119] or extracellular isoforms IV [120,121] and IX [122,123]. Whether variations in the expression patterns or levels of carbonic anhydrases generate asymmetry in MCT transport remains to be investigated.

## 5. Conclusions

Intrinsic asymmetry in transmembrane facilitation by channels and secondary active transporters is a reality. It is probably much more common than currently expected. The degree by which the transmembrane substrate equilibrium position is shifted may be smaller than what is achievable by primary active ATPases, but physiological relevance has been shown, including severe effects on tumor malignancy.

The underlying principles for biased transport are based on asymmetric properties of the transporting entity itself or conferred by interaction partners (proteins or small molecules). Such properties include distinct surface charges, substrate affinities, open probabilities, and coupled chemical reactions on the extra- and intracellular sides. Even though the determination of undisturbed import/export kinetics or the equilibrium position under steady-state conditions is demanding, it is a worthwhile endeavor that will contribute to the basic understanding of physiological and pathophysiological situations.

## Data Availability

No new data were created or analyzed in this study.

## Abbreviations

The following abbreviations are used in this manuscript:

p*K*a	Negative logarithm of the acid dissociation constant
MIP	Major intrinsic protein superfamily
MFS	Major facilitator superfamily
Kir	Inward-rectifying potassium channel
EAAC1	Excitatory amino acid carrier 1
Lyp1	Yeast lysine permease 1
SMCT	Sodium-coupled monocarboxylate transporter
AQP	Aquaporin
ACR3	Arsenic compound resistance 3
FNT	Formate-nitrite transporter
MCT	Monocarboxylate transporter
CA	Carbonic anhydrase
